# Gold Nanoparticles Promote Oxidant-Mediated Activation of NF-*κ*B and 53BP1 Recruitment-Based Adaptive Response in Human Astrocytes

**DOI:** 10.1155/2015/304575

**Published:** 2015-06-23

**Authors:** Jennifer Mytych, Anna Lewinska, Jacek Zebrowski, Maciej Wnuk

**Affiliations:** ^1^Department of Genetics, University of Rzeszow, Rejtana 16C, 35-959 Rzeszow, Poland; ^2^Department of Biochemistry and Cell Biology, University of Rzeszow, Zelwerowicza 4, 35-601 Rzeszow, Poland; ^3^Department of Plant Physiology, University of Rzeszow, Werynia 502, 36-100 Kolbuszowa, Poland

## Abstract

Nanogold-based materials are promising candidate tools for nanobased medicine. Nevertheless, no conclusive information on their cytotoxicity is available. In the present study, we investigated the effects of gold nanoparticles (AuNPs) on human astrocytes *in vitro*. Nanogold treatment in a wide range of concentrations did not result in cytotoxicity. In contrast, nanogold provoked changes in the astrocyte cell cycle and induced senescence-associated *β*-galactosidase activity. AuNPs promoted oxidative stress and caused activation of NF-*κ*B pathway. After nanogold treatment, an inverse correlation between the formation of 53BP1 foci and micronuclei generation was observed. The robust 53BP1 recruitment resulted in reduced micronuclei production. Thus, nanogold treatment stimulated an adaptive response in a human astrocyte cell.

## 1. Introduction

Gold nanoparticles (AuNPs) of high chemical stability, well-controlled size, and surface functionalization are considered promising tools for nanobased medicine, including biosensors, bioimaging, photothermal therapy, and targeted drug delivery [1–3]. The delivery of therapeutic biomolecules and transgenes is particularly challenging in the central nervous system (CNS) due to blood-brain barrier (BBB) formed by endothelial cells, pericytes, astrocytes, and microglial cells, which can be to some extent overcome by the use of nanogold facilitating the treatment and diagnosis of neurological disorders [3–6]. Glucose-coated gold nanoparticles can selectively cross human brain endothelium* in vitro* and localize in astrocytes [5]. Although the gold core is inert and nontoxic and AuNPs are thought to be highly biocompatible [7], they may also interact with biological material and have biological effects, which needs to be carefully addressed [8, 9].

Conflicting results on nanogold-mediated toxicity in diverse mammalian cells* in vitro* have been reported [7, 10–18]. 1.4 nm AuNPs were found to be much more toxic than 15 nm AuNPs to connective tissue fibroblasts, epithelial cells, macrophages, and melanoma cells [10]. In contrast, 18 nm AuNPs were not toxic to human leukemia cells [7] and 60 nm AuNPs were not toxic to murine macrophages [11]. As the response to nanogold is affected by the nanoparticle size and the cell type examined, the effects of AuNPs should be also evaluated in every cell type to be used for nanogold-based technologies, especially in the brain.

Data on nanoparticle effects in the brain tissue and cells both* in vitro* and* in vivo* are scarce and two main features were observed, namely, nanoparticle-mediated oxidative stress and inflammatory response [19–23]. A mixed primary cell model consisting mainly of neurons and astrocytes and a minor proportion of oligodendrocytes was used to study nanoparticle-mediated neurotoxicity [22]. In contrast to nontoxic 20 nm gold nanoparticles, 20 nm silver nanoparticles were found to be effective against primary mixed neural cell cultures [22]. AgNPs (up to 20 *μ*g/mL) stimulated calcium dysregulation and reactive oxygen species (ROS) formation-based response [22]. Moreover, astrocytes were found to be much more sensitive to nanosilver treatment compared with neurons and AgNPs were mainly taken up by astrocytes and not by neurons [22].

As available information on nanogold-mediated effects on a human astrocyte cell is limited, we decided to evaluate AuNP-associated response in human astrocytes* in vitro*; we especially focused on AuNP effects on cyto- and genotoxicity, as well as nanogold-induced oxidative stress and NF-*κ*B pathway.

## 2. Materials and Methods

### 2.1. Chemicals

Gold nanoparticles (AuNPs) were purchased from Sigma (765449, 5 nm diameter, carboxylic acid functionalized, PEG 5000 coated, OD = 50, and dispersion in H_2_O, Poznan, Poland) and phosphate-buffered saline (PBS) was obtained from Gibco, Invitrogen Corporation, Grand Island, NY, USA. All other reagents, if not mentioned otherwise, were purchased from Sigma (Poznan, Poland) and were of analytical grade.

### 2.2. Characterization of AuNPs: AFM

Commercially available gold nanoparticles (765449, Sigma) were characterized using atomic force microscopy (AFM). The shape, size distribution, tendency to agglomerate, and concentration in suspension of AuNPs were evaluated using Bioscope Catalyst II atomic force microscope equipped with Nanoscope V controller (Veeco, Santa Barbara, CA, USA). AuNPs were suspended in distilled water and the suspension was spread out over the surface of freshly cleaved mica (V1 grade, Ted Pella Inc., USA). The particles were then allowed to adhere to the mica surface as a result of drying out under gentle N_2_ stream. Topography AFM imaging of the preparations with deposited gold nanoparticles was carried out in the Peak Force Tapping mode (amplitude set point 100 nN, gain 0.8) using Bruker's sharp silicon nitride SNL-10 A probes of nominal spring constant *k* = 0.35 N/m and 2 nm nominal tip radius. Height sensor and Peak Force error images were collected at the scan rate of 0.35 Hz and at the resolution of 768 pixels per line using Nanoscope (v. 8.15sr3, Bruker) software. Images were processed and analyzed for the particle height and the size distribution by means of Nanoscope Analysis (v. 1.50, Bruker Corporation, Germany) software. The concentration of AuNPs in suspension was also evaluated using atomic force microscopy. A drop (1 *μ*L) of the nanoparticle suspension was added to a drop (1 *μ*L) of 10% Tween-20 previously deposited on freshly cleaved piece of mica. As a result of the fusion of both portions of liquids, the suspension with reduced surface tension easily spread over the mica substrate allowing for approximately equal deposition of the nanoparticles throughout mica surface area (c. 100 mm^2^). The nanoparticles were allowed to deposit on the mica substrate during subsequent drying at nitrogen atmosphere and occasional gentle swaying. The preparations with adhered AuNPs were then imaged using AFM. The height sensor images were collected for surface area of 10 *μ*m × 10 *μ*m taken at 4 random locations for two different samples. The images were processed using Nanoscope Analysis software (v. 1.50, Bruker) and exported as TIFF-extension files for the particle counting with ImageJ v. 1.45 software. The amount of detected particles multiplied by the ratio of the mica sheet area to the real area of the image was considered as the estimation of the nanoparticle number in the drop of applied nanoparticle suspension.

### 2.3. Cell Culture

Gibco Human Astrocytes were obtained from Life Technologies (N7805-100, Warsaw, Poland). The cells were cultured at 37°C in Gibco Astrocyte Medium (A1261301, Dulbecco's Modified Eagle's Medium (DMEM), N-2 Supplement, and One Shot Fetal Bovine Serum (FBS)) in a humidified atmosphere in the presence of 5% CO_2_. According to the manufacturer's instructions, Geltrex matrix-coated plates (A14132) and 3000 cells per well in a 96-well plate or 5000 cells per well in a 4-well plate were used. Astrocytes were cultured for 24 h, medium was then discarded and fresh medium containing 1.1 × 10^9^, 1.1 × 10^10^, 1.1 × 10^11^, and 5.5 × 10^11^ AuNPs/mL that corresponds to 1.4, 14, 140, and 700 ng/mL [17] was added and cells were cultured for another 96 h. Every 48 h, the medium supplemented with AuNPs was replaced by a fresh one.

### 2.4. Cytotoxicity, Cell Cycle Analysis, and SA-*β*-gal Activity

After 96 h treatment with gold nanoparticles, acridine orange-ethidium bromide staining was used to assess cytotoxicity [24]. Cell cycle analysis was conducted using an In Cell Analyzer 2000 (GE Healthcare, UK) equipped with a high performance CCD camera [25]. Senescence-associated *β*-galactosidase activity (SA-*β*-gal) was measured according to Mytych et al. [26].

### 2.5. Oxidative Stress

After 96 h treatment with gold nanoparticles, the steady-state level of reactive oxygen species (ROS) inside a cell was measured using redox-sensitive fluorogenic probe 2′,7′-dichlorodihydrofluorescein diacetate (H_2_DCF-DA) and imaging cytometry (In Cell Analyzer 2000 equipped with a high performance CCD camera, GE Healthcare, UK). Briefly, the cells were incubated in PBS containing 5 *μ*M H_2_DCF-DA for 15 min in the dark, cells were then washed, and intracellular fluorescent signals were acquired and quantified using In Cell Analyzer 2000 Software (GE Healthcare). The level of ROS is presented as relative fluorescence units (RFUs).

### 2.6. NF-*κ*B Activation

After 96 h treatment with gold nanoparticles, immunostaining protocol was used as previously described [26]. Briefly, fixed cells were incubated with a primary antibody anti-p65 (1 : 100) (Abcam, UK) and a secondary antibody conjugated with FITC (1 : 1000) (Pierce, UK). Nuclei were visualized with Hoechst 33342. Digital cell images were captured with an In Cell Analyzer 2000 (GE Healthcare, UK) equipped with a high performance CCD camera. The NF-*κ*B p65 nuclear-positive cells were scored [%].

### 2.7. Micronuclei Production

After 96 h treatment with gold nanoparticles, a BD Gentest Micronucleus Assay Kit with the standard protocol was used [26].

### 2.8. 53BP1 Immunostaining

After 96 h treatment with gold nanoparticles, immunostaining protocol was used as previously described [26]. Briefly, fixed cells were incubated with a rabbit polyclonal antibody against 53BP1 (1 : 200) (Novus Biologicals, Poland) and with a FITC-conjugated, secondary polyclonal antibody against rabbit IgG (1 : 200) (BD Biosciences, Germany). Nuclei were visualized with Hoechst 33342. Digital cell images were captured with an In Cell Analyzer 2000 (GE Healthcare, UK) equipped with a high performance CCD camera. The cells with 0, 1, 2, 3, and more than 3 53BP1 foci were scored [%].

### 2.9. Statistical Analysis

The results represent the mean ± SD from at least three independent experiments. Statistical significance was assessed by 1-way ANOVA using GraphPad Prism 5, with Dunnett's multiple comparison test.

## 3. Results

**Figure 1:**
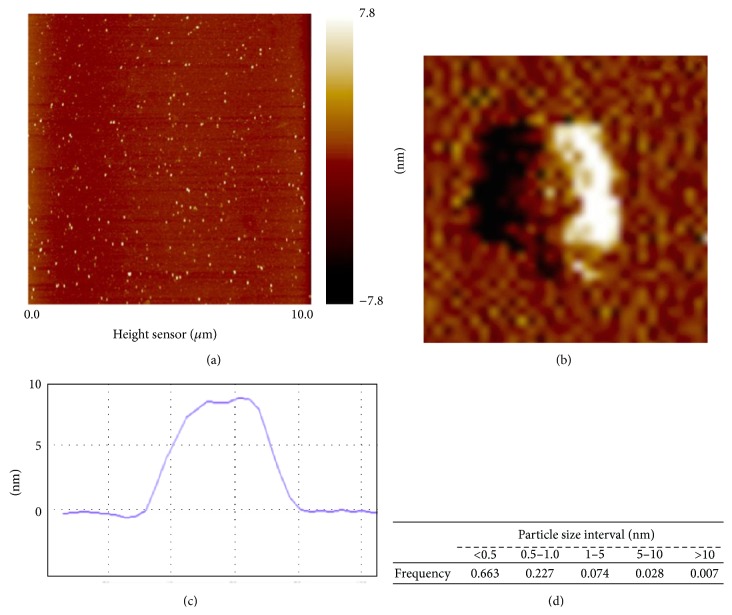
Characteristics of gold nanoparticles (AuNPs) using atomic force microscopy (AFM). (a) Representative height sensor image of AuNPs deposited on nonfunctionalized mica showing their diversity in size and lack of the tendency for particle agglomeration in water suspension. (b) Typical shape of a gold nanoparticle obtained in Peak Force mode at high-resolution imaging (the particle height equal to c. 9.5 nm). (c) A height profile of an individual AuNP used for determination of the particle size. (d) The distribution of nanoparticle size.

As gold nanoparticles were commercially purchased, AuNPs were characterized for selected physical properties before the analysis of their effects on human astrocytes. Atomic force microscopy (AFM) imaging showed that AuNPs suspended in water were capable of dispersing on mica substrate without tendency for agglomerating (Figure 1(a)). This property was likely to be associated to some extent with commercial functionalizing of the AuNP with polyethylene glycol (PEG).

The AFM images also showed the high purity of AuNP samples, as no contamination with microsized components has been detected. The gold nanoparticles showed rather irregular than spherical shape as visualized at high resolution in the Peak Force Tapping mode (Figure 1(b)). The cross-sectional height profile on *z*-axis (Figure 1(c)) was the base for determination of the individual particle size at the resolution below 0.3 nm. The frequency of AuNP size distribution for a representative particle sample (Figure 1(d)) indicated rather high dispersion in the particle size below 10 nm with large prevalence of fractions below 1 nm in size. The estimated AuNP concentration was approximately 1.1 × 10^11^ particles/*μ*L (1.1 × 10^14^ particles/mL). Moreover, we have compared our AFM-based calculations with absorption cross section-based calculations using the formula

(1) $$\begin{array}{c} \text{OD}=\log\left(\;e\;\right)\times \text{absorption cross section}\times \left(\;\frac{N}{V}\;\right)\times l, \end{array}$$

where log(*e*) = 0.434568904, absorption cross section for 5 nm AuNPs is 6 × 10^−14^ cm^2^, *N*/*V* is the number of nanoparticles per volume, and *l* is the length of the cuvette, 1 cm.

We have obtained that *N*/*V* = 1.9 × 10^15^ particles/mL that differs from that obtained after AFM-based calculations (1.1 × 10^14^ particles/mL). As we have already detected a large fraction of AuNPs of diameter lower than 0.5 nm in the solution, such discrepancies are not so surprising. We believe that our AFM-based calculations are more accurate and adequate in this particular case.

**Figure 2:**
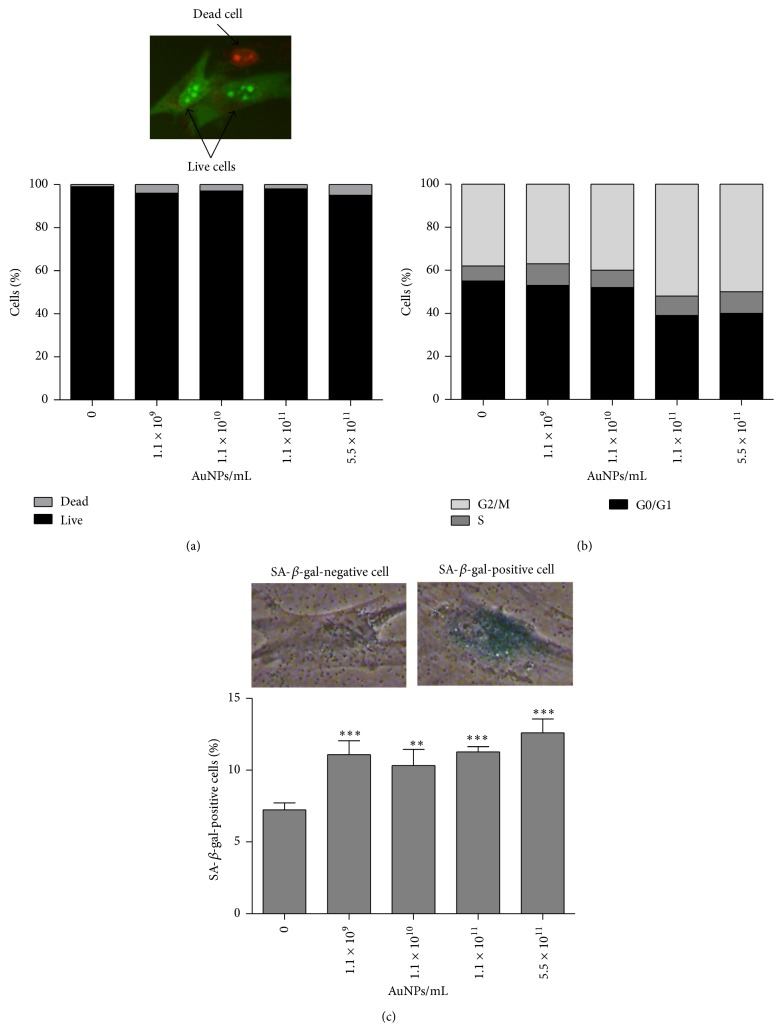
Nanogold-mediated cytotoxicity (a), changes in the cell cycle (b), and ability to induce stress-induced premature senescence (SIPS) (c). Human astrocytes were treated with 1.1 × 10^9^–5.5 × 10^11^ AuNPs/mL for 96 h. (a) Cell viability was assessed using acridine orange-ethidium bromide staining. Arrows indicate live cells (green) and a dead cell (red). (b) Cell cycle analysis using an In Cell Analyzer 2000 (GE Healthcare, UK). (c) SIPS was assessed as SA-*β*-gal activity. The bars indicate the SD, *n* = 3, ^*∗∗∗*^ *P* < 0.001, and ^*∗∗*^ *P* < 0.01 compared with control (ANOVA and Dunnett's* a posteriori* test). Typical micrographs showing a SA-*β*-gal-positive cell and a SA-*β*-gal-negative cell are also presented.

As nanogold in a range of concentrations from 36 to 1000 ng/mL was screened for cytotoxic effects in different mammalian cell lines [14], we used AuNP concentrations ranging from 1.4 to 700 ng/mL that corresponds to 1.1 × 10^9^–5.5 × 10^11^ AuNPs/mL [17]. As a negative control, supernatant of AuNP after centrifugation was used. We were not able to observe any differences compared to control conditions (data not shown). Cytotoxic potential of AuNPs was minimal as estimated using acridine orange-ethidium bromide staining (Figure 2(a)).

AuNP treatment resulted in astrocyte cell death in up to 5% of total population examined (Figure 2(a)). In contrast, nanogold promoted changes in the astrocyte cell cycle (Figure 2(b)). After AuNP treatment, cells preferentially accumulated in the G2/M phase of the cell cycle. The percentage of cells in the G2/M phase of the cell cycle increased from 38% (control conditions) to approximately 52% (treatment with 1.1 × 10^11^ AuNPs/mL) and 50% (treatment with 5.5 × 10^11^ AuNPs/mL) (Figure 2(b)). Moreover, nanogold caused an increase in the level of SA-*β*-gal-positive cells, which may suggest that AuNP may stimulate stress-induced premature senescence (SIPS) in human astrocytes (Figure 2(c)). Treatments with 1.1 × 10^11^ AuNPs/mL and 5.5 × 10^11^ AuNPs/mL resulted in 55% and 75% increase in SA-*β*-gal-positive cells compared with control, respectively, *P* < 0.001 (Figure 2(c)).

**Figure 3:**
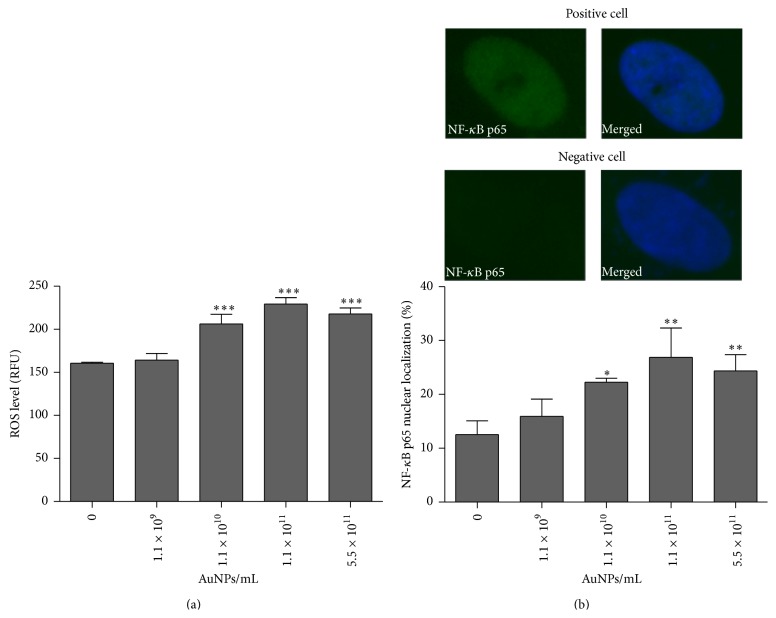
Nanogold-induced oxidative stress (a) and NF-*κ*B activation (b). Human astrocytes were treated with 1.1 × 10^9^–5.5 × 10^11^ AuNPs/mL for 96 h. (a) The steady-state level of reactive oxygen species (ROS) was measured using 2′,7′-dichlorodihydrofluorescein diacetate (H_2_DCF-DA) and imaging cytometry. The level of ROS is presented as relative fluorescence units (RFUs). The bars indicate the SD, *n* = 3, ^*∗∗∗*^ *P* < 0.001 compared with control (ANOVA and Dunnett's* a posteriori* test). (b) After AuNP treatment, NF-*κ*B p65 was translocated into nucleus (green). Nuclei were visualized with Hoechst 33342 (blue). The bars indicate the SD, *n* = 3, ^*∗∗*^ *P* < 0.01, and ^*∗*^ *P* < 0.05 compared with control (ANOVA and Dunnett's* a posteriori* test).

Nanogold also induced oxidative stress in human astrocytes (Figure 3(a)).

The level of reactive oxygen species (ROS) was increased by 40% and 34% after treatments with 1.1 × 10^11^ AuNPs/mL and 5.5 × 10^11^ AuNPs/mL compared with control, respectively, *P* < 0.001 (Figure 3(a)). AuNPs also provoked NF-*κ*B activation because p65 nuclear signals were elevated after gold nanoparticle treatment (Figure 3(b)). Treatments with 1.1 × 10^11^ AuNPs/mL and 5.5 × 10^11^ AuNPs/mL caused approximately 2-fold increase in p65 nuclear signals compared with control, *P* < 0.01 (Figure 3(b)).

**Figure 4:**
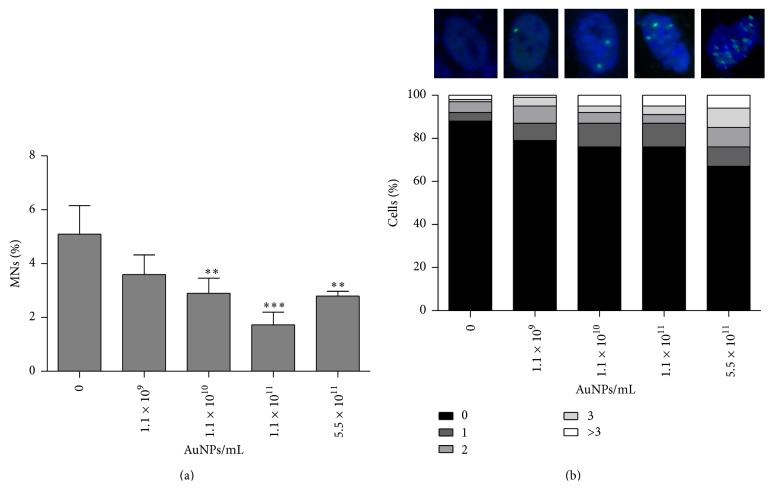
The effect of nanogold on micronuclei production (a) and 53BP1 recruitment (b). Human astrocytes were treated with 1.1 × 10^9^–5.5 × 10^11^ AuNPs/mL for 96 h. (a) The cytokinesis-block micronucleus (CBMN) assay. The bars indicate the SD, *n* = 3, ^*∗∗∗*^ *P* < 0.001, and ^*∗∗*^ *P* < 0.01 compared with control (ANOVA and Dunnett's* a posteriori* test). (b) 53BP1 foci were revealed using 53BP1 immunostaining. Cells with 0, 1, 2, 3, and more than 3 53BP1 foci were scored [%].

Nanogold did not stimulate micronuclei production (Figure 4(a)).

In contrast, the level of binucleated cells with micronuclei dropped after AuNP treatment, *P* < 0.01 and *P* < 0.001 (Figure 4(a)). We also investigated AuNP-mediated formation of p53 binding protein (53BP1) foci, which are considered to be accumulated at site of double strand breaks (DSBs) being a part of DNA repair process. Surprisingly, an inverse correlation between 53BP1 foci and micronuclei generation was observed (Figure 4(b)). Treatments with 1.1 × 10^11^ AuNPs/mL and 5.5 × 10^11^ AuNPs/mL caused a 2- and 2.75-fold increase in the formation of 53BP1 foci compared with control, respectively (Figure 4(b)).

## 4. Discussion

As data on nanoparticle (NP) effects in the brain, especially AuNP action on human astrocytes, are limited [21, 22], we decided to investigate astrocyte response to nanogold treatment. A range of AuNP concentrations from 1.4 to 700 ng/mL (1.1 × 10^9^–5.5 × 10^11^ particles/mL) was selected on the basis of previously published results on AuNP-mediated toxic effects on diverse mammalian cell lines, namely, PK-15 (porcine kidney), Vero (African green monkey kidney), NIH3T3 (mouse embryonic fibroblast), and MRC5 (human normal lung fibroblast) cells [14]. Nanogold-associated adaptive response without cytotoxicity was observed, which was mediated by increased ROS levels, activation of NF-*κ*B pathway, and robust 53BP1 recruitment resulting in genomic stability.

Our data on limited AuNP cytotoxicity against astrocyte cells are in agreement with previously reported results using primary mixed neural cell culture as a model [22]. Nanogold when used up to 100 *μ*g/mL did not provoke toxic effects against neurons and astrocytes [22]. Moreover, AuNPs (10^9^ particles/mL) were not cytotoxic and did not induce apoptotic cell death in N9 murine microglia and SH-SY5Y human neuroblastoma cells [21]. In contrast, nanogold affected the astrocyte cell cycle leading to the arrest at the G2/M phase of the cell cycle, which may reflect a stress response. Nanogold (≥180 ng/mL) also caused a significant delay of the G2/M phase of lung fibroblast cell cycle [14]. The inhibition of cell proliferation without causing cell death may allow the cell to alleviate the effects of stress stimuli. Indeed, the suppression of MRC5 cell proliferation and resistance to nanogold-induced cyto- and genotoxicity was mediated by the activation of pathways involved in DNA damage response and repair, cell cycle regulation, and redox homeostasis, as well as ABC transporters [14].

Nanogold also induced senescence-associated *β*-galactosidase activity in human astrocytes, which is a sign of stress-induced premature senescence (SIPS) and may contribute to AuNP-mediated inhibition of cell proliferation [27]. More recently, nanodiamond powder was also shown to be an inducer of SIPS in human cervical cancer cells [26].

Nanogold stimulated reactive oxygen species (ROS) production in human astrocytes, which may be both related and not related to AuNP-induced cellular senescence. Indeed, the role of ROS in the cellular metabolism is much more complex than previously thought [28, 29]. Of course, when the level of ROS is high, the impairment of redox homeostasis may lead to oxidative protein and DNA damage and a concomitant apoptotic/necrotic cell death. However, ROS at the moderate levels are considered molecular secondary messengers regulating cellular signaling pathways [30]. ROS may modulate redox reactions affecting active sites of enzymes and the activity of transcription factors, such as NF-*κ*B, JUN, and FOS [31]. After treatment with 20 *μ*g/kg body weights of gold nanoparticles for 3 days, the levels of lipid peroxidation and oxidative DNA damage, and Hsp70, IFN-*γ*, and caspase 3 were increased, whereas the activity of glutathione peroxidase was decreased in rat brain [23]. The authors concluded that nanogold treatment may result in inflammation and DNA damage/cell death [23]. Nanogold (20 *μ*g/mL) and nanosilver (20 *μ*g/mL) stimulated ROS production in neurons and astrocytes, but the effect of AgNPs was 5-fold stronger than the effect of AuNPs and contrarily to AgNPs, AuNPs did not provoke cytotoxicity [22]. AuNPs also decreased reduced glutathione (GSH) pools in N9 murine microglia and SH-SY5Y human neuroblastoma cells without causing cytotoxicity and apoptosis [21]. As increased ROS level was not accompanied by astrocyte cell death, we decided to evaluate whether nanogold-associated imbalanced redox homeostasis may affect redox-sensitive transcription factor NF-*κ*B. Indeed, AuNP treatment resulted in increased nuclear p65 signals, which is in agreement with the view that NF-*κ*B is the sensor of oxidative stress and its regulation is redox-based [32, 33]. NF-*κ*B is a dimeric transcription factor composed of different members of the Rel family, such as p65 (RelA), p50, p52, c-Rel, and RelB and the mammalian NF-*κ*B protein family includes five members: NF-*κ*B1 (p50/p105), NF-*κ*B2 (p52/p100), RelA (p65), RelB, and c-Rel [34]. Upon activation, NF-*κ*B is rapidly released from the complex of NF-*κ*B/NF-*κ*B inhibitor and translocated into the nucleus where a group of NF-*κ*B-responsive effector genes involved in stress responses, inflammation, cell proliferation, and apoptosis can be regulated [35]. It is believed that NF-*κ*B activation promotes cell survival and exerts antiapoptotic effects [36, 37]. More recently, AuNPs were found to be an activator of NF-*κ*B in murine B lymphocyte cell line (CH12.LX) [38]. Nanogold induced activation of the canonical NF-*κ*B signaling pathway as evidenced by I*κ*B*α* phosphorylation at serine residues 32 and 36 followed by I*κ*B*α* degradation and increased nuclear RelA (p65), which, in turn, resulted in altered B lymphocyte function (i.e., increased antibody expression) [38].

Nanogold was reported to promote genotoxicity and DNA damage response in different cell types* in vitro* and* in vivo* [14, 23, 39, 40]. In the AuNP treated lung fibroblasts (72 h exposure time, 1 nM AuNPs), genotoxic events were observed and the level of several proteins was affected including oxidative stress related proteins (NADH ubiquinone oxidoreductase (NDUFS1), protein disulfide isomerase associate 3 (PDIA3), heterogeneous nuclear ribonucleoprotein C1/C2 (hnRNP C1/C2), and thioredoxin-like protein 1 (TXNL1)), as well as proteins associated with cell cycle regulation, cytoskeleton, and DNA repair (heterogeneous nuclear ribonucleoprotein C1/C2 (hnRNP C1/C2) and secernin-1 (SCN1)) [40]. The authors concluded that AuNP treatment can induce oxidative stress-mediated genomic instability [40]. More recently, similar genomic response was observed in lung fibroblasts [14]. After 360 ng/mL AuNP treatment, the expression of genes involved in DNA damage response, repair pathways, and redox homeostasis was increased (e.g., the tumor suppressor* p53* and* BRCA1* genes, genes involved in base-excision respire (BER) and homologous recombination pathways, genes associated with mismatch repair and translesion synthesis:* MLH3* and* Rev1*, and genes encoding glutathione reductase, glutathione transferase, and glutaredoxin 2) [14]. However, the authors suggested that such response may contribute to resistance to AuNP-induced cyto- and genotoxicity [14]. Data on nanogold-induced genotoxic stress in human astrocytes are lacking. We showed for the first time that AuNPs did not provoke genotoxicity in human astrocytes by means of the cytokinesis-block micronucleus (CBMN) assay. In contrast, the robust recruitment of 53BP1 was observed. During cellular response to DNA damage, p53 binding protein- (53BP1-) dependent pathway is activated: 53BP1 is recruited to sites of DNA damage due to methylation state-specific recognition of histone H4-K20 by 53BP1 [41]. 53BP1 was shown to be involved in the regulation of activation of the G2/M phase checkpoint, the intra-S phase checkpoint, and repair of DNA double strand breaks (DSBs)* via* nonhomologous end-joining (NHEJ) [42–45]. A link between recruitment of 53BP1 and resolution of DNA damage has been previously established [46]. In 53BP1-depleted WI38 human fibroblasts exposed to agents causing DNA damage, the fraction of micronuclei-positive cells was elevated [46]. Additionally, enhanced activation or upregulation of 53BP1 resulted in lower level of chromosomal damage as a response to DNA damage [46]. Thus, 53BP1 contributed to genomic stability in human fibroblasts [46]. Perhaps, the robust recruitment of 53BP1 may also result in reduced micronuclei production after astrocyte treatment with gold nanoparticles.

In conclusion, we showed for the first time that nanogold may trigger adaptive response in human astrocytes, which was mediated by oxidant-based activation of NF-*κ*B and 53BP1 recruitment promoting cell survival and resistance to nanogold-mediated genotoxicity.
